# SST subseasonal-to-seasonal forecasting: A heterogeneous three-path fusion model with wavelet decomposition

**DOI:** 10.1371/journal.pone.0351923

**Published:** 2026-07-07

**Authors:** Xiao Chen, Yihao Wang, Jing Chen, MingXin Liu

**Affiliations:** 1 Research Center for Marine Science, Hebei Normal University of Science and Technology, Qinhuangdao, China; 2 Hebei Key Laboratory of Ocean Dynamics, Resources and Environments, Qinhuangdao, China; 3 College of Mathematics and Computer Science, Guangdong Ocean University, Zhanjiang, China; 4 College of Electronic and Information Engineering, Guangdong Ocean University, Zhanjiang, China; National Taiwan Ocean University, TAIWAN

## Abstract

El Niño-Southern Oscillation (ENSO), the Earth’s dominant mode of interannual climate variability, is closely linked to sea surface temperature (SST) variability in the central and eastern equatorial Pacific. Accurate subseasonal-to-seasonal SST forecasting in this region supports ocean monitoring and environmental assessment. We propose WDFormer (Wave Decomposition Former), a daily SST forecasting model built on a “decomposition, multi-path processing, and adaptive fusion” paradigm. The model uses 365 daily SST observations from local 4×4 grids around representative ENSO monitoring stations to predict daily SST values over 7–90-day horizons. WDFormer first applies wavelet decomposition to separate the input sequence into a smoother low-frequency component and a higher-frequency residual. Lightweight MLP-based branches process the original and low-frequency components, while a Transformer-based branch models the residual dynamics. Outputs are integrated through a learnable gating mechanism. Experiments on daily OISST data from five representative ENSO subregions show that WDFormer outperforms several deep learning baselines at most 30–90-day horizons. Comparisons with persistence and daily climatology confirm that persistence dominates at very short lead times, whereas WDFormer provides clearer advantages as the horizon extends. An ONI-based ENSO-phase and El Niño-stage stratified evaluation shows that WDFormer achieves the lowest RMSE under El Niño, La Niña, and Neutral conditions, as well as during El Niño onset/development, mature/peak, and decay/termination stages for the 90-day task. Bootstrap confidence intervals and paired RMSE comparisons further support the robustness of the 90-day results. These results support decomposition-based heterogeneous modeling for daily SST forecasting at 30–90-day horizons and motivate broader extensions to probabilistic and multi-variable forecasting.

## Introduction

Sea surface temperature (SST) in the equatorial eastern Pacific is a key surface expression of the El Niño-Southern Oscillation (ENSO), the Earth’s dominant mode of interannual climate variability [1–5]. Sustained anomalous warming (El Niño) or cooling (La Niña) in this region for five or more consecutive months is commonly used to identify ENSO events [6], which can propagate via teleconnections and modulate large-scale atmospheric circulation systems [7,8]. SST variability is also embedded in the broader context of ocean heat uptake and climate-related extreme events [9,10]. Recent studies further show that oceanic and local land–atmosphere conditions can contribute to extreme regional heat events, underscoring the importance of monitoring slowly varying oceanic boundary conditions [11]. Accurate short-lead daily SST forecasting in the equatorial Pacific can therefore support ocean monitoring, subseasonal environmental assessment, and event-conditioned analysis [12,13].

Current SST forecasting methods fall into two broad categories: mechanism-based numerical models [14–16] and data-driven approaches. Hybrid and intermediate coupled models have long been used for ENSO-related SST prediction, but their performance depends on initialization, physical parameterization, and the representation of air–sea interactions [14,15]. Probabilistic dynamical systems such as the North American Multi-Model Ensemble (NMME) provide important seasonal ENSO guidance, but they differ from the present daily local-grid forecasting setting in initialization, spatial scale, forecast horizon, and verification protocol [16].

Data-driven SST forecasting has developed from conventional neural-network and sequence-learning methods to more recent attention-based architectures. Early neural-network studies showed the potential of data-driven models for tropical Pacific SST forecasting [17]. Recurrent models such as LSTM were then introduced to improve temporal dependency learning [18], and were subsequently applied to SST prediction tasks [19]. CNN–LSTM hybrids and attention-enhanced LSTM models further attempted to combine local feature extraction with sequential modeling for satellite or tropical Pacific SST data [20,21]. More recently, Transformer-based models have used self-attention to capture long-range dependencies in time series [22–25]. These models provide strong sequence representations, but they often process mixed-frequency SST signals within a unified architecture.

Despite these advances, daily SST forecasting at 30–90-day horizons remains challenging. Three key gaps motivate this work. First, many data-driven studies emphasize short horizons (≤15 days), and accuracy often degrades as the lead time extends. Second, SST is a complex superposition of intraseasonal, seasonal, interannual, and lower-frequency signals alongside stochastic noise; applying a single monolithic network to this mixed-frequency sequence can lead to over-smoothing of slowly varying components and underfitting of high-frequency residuals [26]. Third, although Transformer-based models capture multi-scale dependencies well, their attention computation can be costly for long input windows, and the resulting predictions may suffer from trend drift if low-frequency and residual dynamics are not explicitly separated [23,27].

To address these gaps, the “decompose first, model later” paradigm has emerged as a promising strategy. Autoformer introduces decomposition into the Transformer framework, DLinear shows that simple decomposition-based linear models can be highly competitive, and TimeMixer further explores decomposable multi-scale mixing for time-series forecasting [26–28]. For SST-related tasks, STL-based decomposition has also been used to separate trend and residual information before forecasting [29]. Building on this line of work, we propose **WDFormer** (Wave Decomposition Former), a heterogeneous three-path fusion model for daily SST forecasting in ENSO monitoring regions. WDFormer uses wavelet decomposition to separate the input sequence into frequency-informed smoother and residual components, then processes each component with a specialized branch matched to its statistical properties.

The main contributions are:

1. **A wavelet-based heterogeneous three-path framework.** WDFormer combines wavelet-based signal separation with component-specific parallel processing, providing a structured alternative to monolithic temporal models for daily SST forecasting.
2. **Component-specific modeling.** Lightweight MLP-based branches handle the original and low-frequency components, while a Transformer branch handles the residual. This design matches model capacity to component complexity while limiting computational overhead.
3. **Empirical evaluation under matched protocols.** WDFormer is compared with five deep learning baselines, plus persistence and daily climatology evaluated on the same test windows. We further provide ablation studies, look-back sensitivity analysis, bootstrap confidence intervals, paired RMSE comparisons, and ONI-based ENSO-phase and El Niño-stage stratified evaluations.

## Materials and methods

### Data and study area

We use the NOAA Optimum Interpolation Sea Surface Temperature (OISST) V2 dataset, which provides globally gridded daily mean SST at 0.25° spatial resolution. The dataset spans January 1, 1991 to December 31, 2024. All experiments use the past 365 days of daily observations as input to forecast future daily SST at horizons of 7, 15, 30, 60, and 90 days.

**Fig 1:**
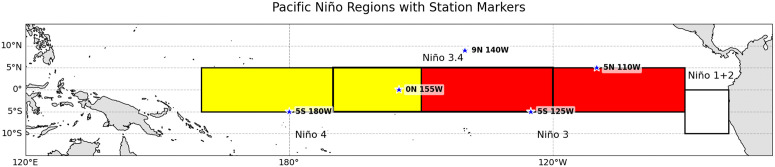
Niño monitoring regions and selected buoy stations. The map was created by the authors using Natural Earth public-domain vector data.

ENSO monitoring primarily encompasses four regions: Niño 1 + 2 (0°–10°S, 85°–90°W), Niño 3 (5°S–5°N, 150°–90°W), Niño 4 (5°S–5°N, 160°E–150°W), and Niño 3.4 (5°S–5°N, 170°–120°W). We select five representative buoy stations covering the Niño 3, Niño 4, and Niño 3.4 regions (Table 1, Fig 1). For each station, a 4×4 grid of surrounding grid points yields 16 features per time step. The dataset is split chronologically into training, validation, and test sets at a 7:2:1 ratio. This corresponds approximately to training from 1991-01-01 to 2014-10-19, validation from 2014-10-20 to 2021-08-08, and testing from 2021-08-09 to 2024-12-31; look-back overlap is used only to construct input sequences.

**Table 1:** Selected buoy stations and coordinates.

No.	Station	Latitude range	Longitude range
1	9N140W	8.5°N–9.5°N	139.5°W–140.5°W
2	5N110W	4.5°N–5.5°N	109.5°W–110.5°W
3	0N155W	0.5°S–0.5°N	154.5°W–155.5°W
4	5S125W	4.5°S–5.5°S	124.5°W–125.5°W
5	5S180W	4.5°S–5.5°S	179.5°W–180.5°W

### WDFormer architecture

**Fig 2:**
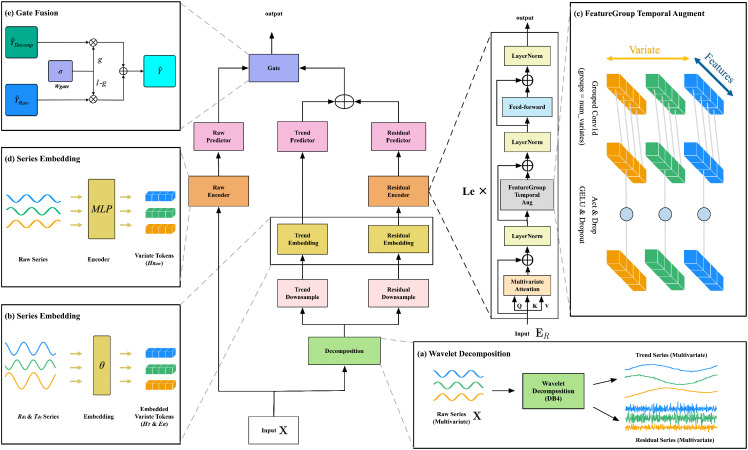
Overall architecture of WDFormer. The input sequence 𝐗 is processed through three parallel paths. **(a)** A wavelet decomposition module separates 𝐗 into trend and residual components. (b, d) The original and trend series are processed by lightweight MLP-based encoders. **(c)** The residual is fed into a Transformer encoder. **(e)** Outputs are adaptively fused by a learnable gating mechanism.

WDFormer is built on a “decomposition, multi-path processing, and adaptive fusion” paradigm comprising four components: (i) a wavelet decomposition module, (ii) three parallel processing paths, and (iii) a gated fusion module. The overall architecture is shown in Fig 2.

### Wavelet decomposition module

Wavelet analysis provides a time–frequency localized representation that is well suited to non-stationary geophysical signals [30]. The Daubechies 4 (db4) wavelet is used as the basis function because of its compact support and regularity [31]. The original SST series 𝐗 is decomposed with a user-adjustable decomposition level *J*. For any time series *x*(*n*):


$$\begin{array}{c} x(n)=\sum_{k}c_{A,J,k}\;\phi_{J,k}(n)+\sum_{j=1}^{J}\sum_{k}c_{D,j,k}\;\psi_{j,k}(n) \end{array}$$ (1)

where $c_{A,J,k}$ are approximation coefficients at level *J*, $\phi_{J,k}(n)$ is the scaling function, $c_{D,j,k}$ are detail coefficients at level *j*, and $\psi_{j,k}(n)$ is the translated mother wavelet.

The *trend component* is reconstructed from the approximation coefficients:


$$\begin{array}{c} T(n)=\sum_{k}c_{A,J,k}\;\phi_{J,k}(n) \end{array}$$ (2)

The *residual component* is:


$$\begin{array}{c} R(n)=x(n)-T(n)=\sum_{j=1}^{J}\sum_{k}c_{D,j,k}\;\psi_{j,k}(n) \end{array}$$ (3)

The low-frequency trend reflects slowly varying background SST and seasonal-scale evolution within the input window. The residual captures higher-frequency departures, including intraseasonal variability and short-lived local perturbations. More explicit attribution to specific physical processes (e.g., Kelvin waves, MJO, PDO) would require additional variables and process-oriented diagnostics beyond the scope of this study.

### Trend path

Because the trend component evolves smoothly, it is processed by a lightweight MLP. The trend input $𝐓\in {\mathbb{R}}^{B\times H\times N}$ is first downsampled by 1D average pooling to yield ${𝐓}_{\text{ds}}\in {\mathbb{R}}^{B\times N\times H^{\prime }}$:


$$\begin{array}{c} {𝐓}_{\text{ds}}=\text{AvgPool1d}({𝐓}^{⊺}) \end{array}$$ (4)

A linear embedding then maps ${𝐓}_{\text{ds}}$ to a hidden dimension *D*, and a two-layer MLP predictor generates the trend prediction ${\hat{𝐘}}_{T}\in {\mathbb{R}}^{B\times N\times P}$.

### Residual path

The residual contains high-frequency fluctuations and complex nonlinear dynamics that benefit from the global dependency modeling of a Transformer encoder. The Transformer architecture uses self-attention to represent long-range dependencies without recurrent computation [22], which is useful for modeling irregular residual variability after decomposition. The residual 𝐑 is downsampled, embedded, and processed through $L_{e}$ Transformer layers, producing residual features ${𝐅}_{R}\in {\mathbb{R}}^{B\times N\times D}$ and prediction ${\hat{𝐘}}_{R}$.

### Original path

An additional MLP-based path processes the original, undecomposed sequence 𝐗 directly, producing prediction ${\hat{𝐘}}_{\text{Orig}}$. This path preserves information that may be lost or distorted during decomposition, increasing the model’s robustness.

### Gated fusion

The three path predictions are combined by a learnable scalar gate. This adaptive fusion design allows the model to adjust the relative contribution of the decomposed paths and the raw path rather than imposing a fixed combination: $g=\text{sigmoid}({𝐖}_{\text{gate}})\in (0,1)$:


$$\begin{array}{c} {\hat{𝐘}}_{\text{fused}}=g\cdot ({\hat{𝐘}}_{T}+{\hat{𝐘}}_{R})+(1-g)\cdot {\hat{𝐘}}_{\text{Orig}} \end{array}$$ (5)

### Choice of decomposition method

Wavelet decomposition was selected because it provides a localized time–frequency representation for non-stationary SST sequences and naturally separates the input into complementary low-frequency and high-frequency components. Moving-average decomposition imposes a fixed smoothing scale, while STL requires a prescribed seasonal period. Although these methods are effective in suitable settings, they are less flexible for daily SST sequences containing both seasonal-scale variability and irregular short-term fluctuations. Therefore, WDFormer adopts db4 wavelet decomposition, with the decomposition level *J* used as a tunable hyperparameter. The contribution of decomposition is further examined through the ablation study in which the decomposition module is removed.

### Baseline models

We benchmark WDFormer against five representative recent time-series models: iTransformer [25], PatchTST [24], Crossformer [32], DLinear [26], and TimeMixer [28]. These baselines cover inverted attention across variables, patch-based temporal encoding, cross-dimension attention, decomposition-based linear prediction, and decomposable multi-scale mixing. We did not include every Transformer variant; for example, Informer is discussed as an important long-sequence forecasting model [23], but the selected baselines provide a compact comparison across the main architectural families most relevant to WDFormer. All models use the same 365-day look-back window and are trained with MSE loss. We also include two non-deep-learning reference baselines evaluated on identical test windows:

- **Persistence**: repeats the last observed SST value for all future lead times.
- **Climatology**: predicts the training-period daily mean SST for the corresponding calendar day.

### Evaluation metrics

We use Root Mean Square Error (RMSE) and Mean Absolute Error (MAE), computed over the full prediction horizon *P* and *N* output variables:


$$\begin{array}{c} \text{RMSE}=\sqrt{\frac{1}{P\cdot N}\sum_{i=1}^{P}\sum_{j=1}^{N}(Y_{ij}-{\hat{Y}}_{ij}{)}^{2}} \end{array}$$ (6)

$$\begin{array}{c} \text{MAE}=\frac{1}{P\cdot N}\sum_{i=1}^{P}\sum_{j=1}^{N}|Y_{ij}-{\hat{Y}}_{ij}| \end{array}$$ (7)

Lower values indicate higher accuracy. RMSE is more sensitive to large deviations, while MAE provides a robust measure of average error magnitude.

To quantify the statistical uncertainty of the reported verification scores, we further used non-parametric bootstrap resampling. Forecast samples were resampled with replacement 2,000 times, and RMSE and MAE were recomputed for each bootstrap replicate. The 2.5th and 97.5th percentiles of the bootstrap distribution were used as the 95% confidence interval (CI). For paired model comparisons, the same bootstrap indices were applied to WDFormer and the corresponding baseline model, and the paired RMSE difference was calculated as


$$\begin{array}{c} \Delta RMSE={RMSE}_{baseline}-{RMSE}_{WDFormer}. \end{array}$$ (8)

A positive value of ΔRMSE indicates lower error for WDFormer. This bootstrap analysis evaluates the statistical uncertainty of the performance metrics rather than providing predictive uncertainty for individual forecasts.

## Results

### Comparison with baseline models

Table 2 presents RMSE and MAE for WDFormer and five deep learning baselines across the five datasets at prediction horizons of 7–90 days. WDFormer achieves the best RMSE in most of the 25 scenarios, with its advantage becoming more pronounced at 30–90-day horizons. At short horizons (7–15 days), Crossformer occasionally outperforms WDFormer, suggesting that different architectural inductive biases suit different forecast regimes.

**Table 2:** RMSE and MAE for WDFormer and five deep learning baselines. Best results per scenario are bold; second-best are underlined.

Dataset Horizon		WDFormer		iTransformer		Crossformer		PatchTST		DLinear		TimeMixer	
		RMSE	MAE	RMSE	MAE	RMSE	MAE	RMSE	MAE	RMSE	MAE	RMSE	MAE
9N140W	7	0.238	0.187	0.257	0.204	**0.217**	**0.164**	0.249	0.193	0.289	0.224	0.233	0.178
	15	0.310	0.245	0.317	0.250	**0.287**	**0.222**	0.315	0.246	0.335	0.259	0.299	0.233
	30	**0.354**	**0.278**	0.379	0.304	0.356	0.282	0.384	0.303	0.386	0.299	0.372	0.290
	60	**0.408**	**0.330**	0.471	0.381	0.427	0.346	0.462	0.366	0.453	0.357	0.450	0.360
	90	**0.444**	**0.359**	0.502	0.414	0.450	0.361	0.483	0.390	0.504	0.405	0.484	0.383
5N110W	7	0.329	0.252	0.385	0.297	**0.305**	**0.227**	0.374	0.273	0.441	0.360	0.334	0.250
	15	0.429	0.327	0.464	0.353	**0.419**	**0.320**	0.485	0.361	0.515	0.422	0.453	0.349
	30	**0.495**	**0.377**	0.566	0.436	0.531	0.408	0.570	0.433	0.598	0.493	0.566	0.446
	60	**0.577**	**0.445**	0.678	0.521	0.633	0.480	0.648	0.491	0.713	0.581	0.657	0.513
	90	**0.643**	**0.495**	0.722	0.569	0.706	0.559	0.742	0.553	0.788	0.634	0.741	0.583
0N155W	7	0.323	0.245	0.370	0.280	**0.304**	**0.221**	0.338	0.255	0.426	0.322	0.331	0.252
	15	**0.406**	**0.305**	0.446	0.343	0.424	0.318	0.432	0.328	0.475	0.360	0.410	0.310
	30	**0.462**	**0.354**	0.536	0.404	0.539	0.405	0.553	0.420	0.530	0.406	0.506	0.389
	60	**0.538**	**0.423**	0.658	0.509	0.640	0.503	0.644	0.496	0.598	0.469	0.566	0.442
	90	**0.569**	**0.450**	0.691	0.536	0.769	0.580	0.746	0.590	0.662	0.529	0.746	0.604
5S125W	7	0.251	0.181	0.284	0.213	**0.234**	**0.171**	0.314	0.232	0.371	0.291	0.251	0.184
	15	**0.330**	**0.242**	0.385	0.290	0.338	0.259	0.385	0.284	0.424	0.333	0.390	0.293
	30	**0.379**	**0.282**	0.435	0.330	0.434	0.330	0.491	0.375	0.487	0.387	0.436	0.322
	60	**0.433**	**0.337**	0.541	0.417	0.484	0.380	0.602	0.461	0.571	0.464	0.522	0.392
	90	**0.459**	**0.362**	0.632	0.487	0.585	0.475	0.678	0.516	0.638	0.524	0.571	0.430
5S180W	7	0.234	0.182	0.252	0.197	0.231	0.179	0.247	0.193	0.250	0.199	**0.223**	**0.171**
	15	**0.273**	**0.217**	0.297	0.237	0.340	0.268	0.329	0.261	0.288	0.230	0.299	0.236
	30	**0.327**	**0.262**	0.343	0.276	0.374	0.297	0.403	0.318	0.329	0.266	0.348	0.280
	60	0.397	0.326	0.441	0.358	0.535	0.437	0.526	0.417	**0.386**	**0.308**	0.480	0.383
	90	0.461	0.373	0.504	0.410	0.741	0.593	0.492	0.392	**0.452**	**0.362**	0.581	0.466

Representative comparisons at 90 days: at 5N110W, WDFormer achieves RMSE 0.643 vs. DLinear 0.788 and TimeMixer 0.741; at 5S125W, WDFormer achieves RMSE 0.459 vs. iTransformer 0.632 and PatchTST 0.678. These results suggest that explicitly separating and processing different signal components improves longer-horizon daily SST forecasting.

To assess whether the 90-day advantage of WDFormer was robust under sample resampling, we performed paired bootstrap comparisons aggregated across the five datasets. WDFormer achieved an overall 90-day RMSE of 0.521 (95% CI: 0.515–0.528), lower than iTransformer (0.617), DLinear (0.620), TimeMixer (0.633), PatchTST (0.639), and Crossformer (0.661). The paired RMSE differences were positive for all comparisons, with 95% CIs that did not include zero: 0.096 for iTransformer (95% CI: 0.090–0.102), 0.099 for DLinear (0.090–0.108), 0.112 for TimeMixer (0.106–0.118), 0.118 for PatchTST (0.111–0.124), and 0.139 for Crossformer (0.130–0.148). These results indicate that the 90-day improvement of WDFormer is not driven by a small number of favorable test samples.

### Comparison with reference baselines

Table 3 compares WDFormer with persistence and climatology baselines, averaged across the five stations. Persistence is highly competitive at 7 days (RMSE 0.245 vs. WDFormer 0.275) and nearly identical at 15 days, reflecting strong short-term SST autocorrelation. As the horizon extends, persistence error grows rapidly, and WDFormer provides increasing benefits: 10.7% RMSE reduction at 30 days, 23.2% at 60 days, and 31.9% at 90 days. WDFormer consistently outperforms the climatology baseline at all horizons, indicating that daily climatological means alone cannot capture event-scale SST variability. The full per-station persistence and climatology results are reported in Table 4.

**Table 3:** Average RMSE across five stations for WDFormer, persistence, and climatology. Negative reduction indicates WDFormer performs worse than persistence.

Method	7d	15d	30d	60d	90d
WDFormer	0.275	0.350	**0.403**	**0.471**	**0.515**
Persistence	**0.245**	**0.349**	0.452	0.613	0.757
Climatology	0.906	0.907	0.914	0.922	0.922
WDFormer vs. Persistence	−12.2%	−0.1%	+10.7%	+23.2%	+31.9%

**Table 4:** Full persistence and daily climatology results under the matched testing protocol. Entries are reported as RMSE/MAE.

Persistence					
Station	7d	15d	30d	60d	90d
9N140W	0.208/0.155	0.298/0.220	0.397/0.298	0.560/0.427	0.729/0.559
5N110W	0.290/0.197	0.433/0.291	0.575/0.395	0.786/0.552	0.954/0.675
0N155W	0.296/0.209	0.404/0.294	0.492/0.370	0.608/0.463	0.708/0.536
5S125W	0.232/0.159	0.352/0.248	0.482/0.350	0.718/0.523	0.936/0.682
5S180W	0.199/0.148	0.258/0.195	0.313/0.239	0.390/0.303	0.456/0.352
**Climatology**					
**Station**	**7d**	**15d**	**30d**	**60d**	**90d**
9N140W	0.610/0.496	0.610/0.496	0.612/0.497	0.613/0.496	0.614/0.498
5N110W	0.962/0.784	0.961/0.784	0.969/0.790	0.977/0.797	0.979/0.802
0N155W	1.118/0.947	1.119/0.948	1.129/0.960	1.140/0.972	1.140/0.972
5S125W	0.955/0.846	0.956/0.848	0.962/0.854	0.972/0.865	0.972/0.865
5S180W	0.887/0.773	0.887/0.773	0.898/0.789	0.908/0.800	0.905/0.795

Table 4 reports RMSE and MAE for the persistence and daily climatology baselines at all five stations and all five prediction horizons, evaluated under the matched testing protocol using the same target windows as the deep learning models.

### Visual analysis of predictions

**Fig 3:**
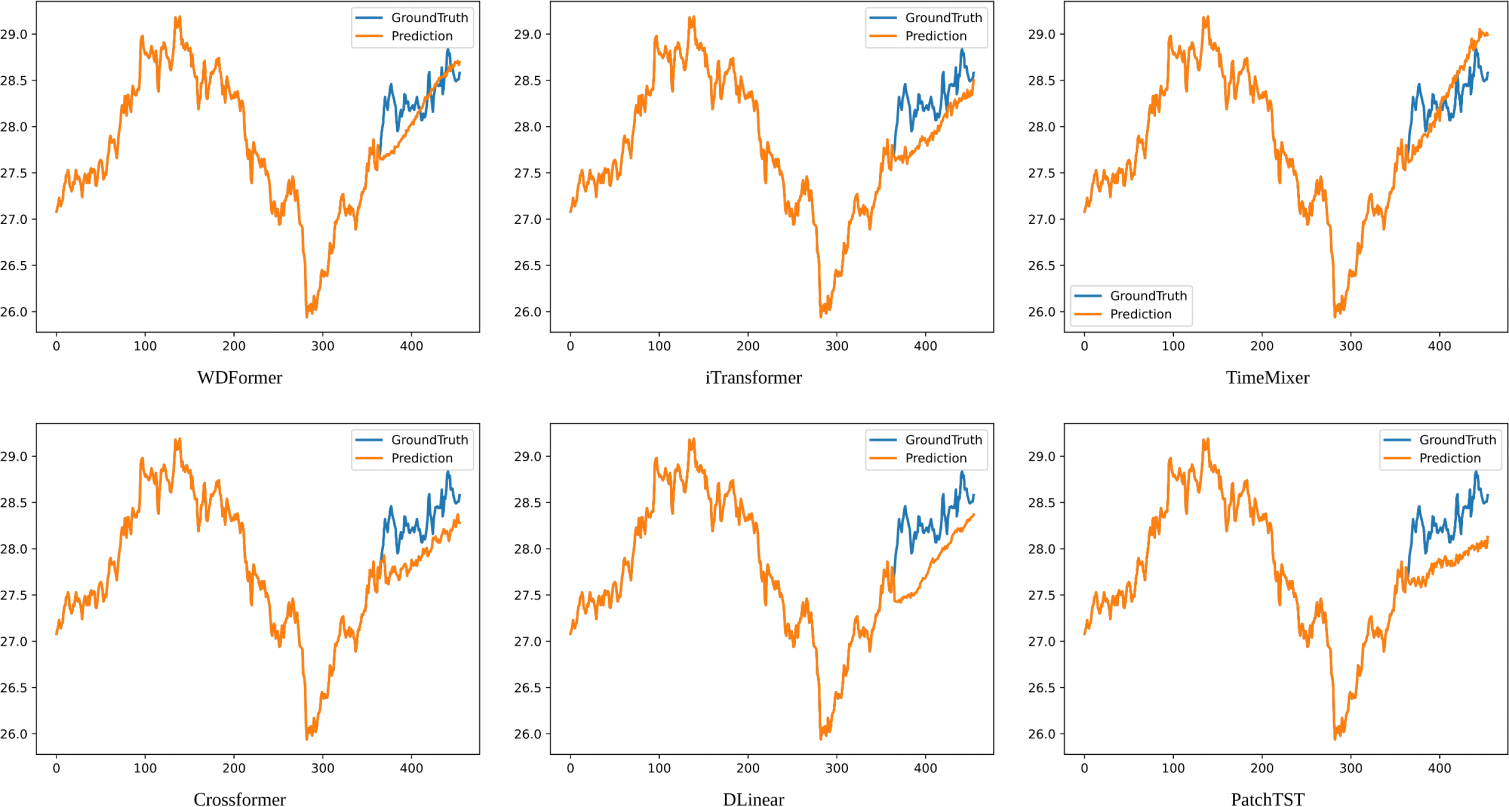
90-day prediction comparison for the 9N140W dataset.

**Fig 4:**
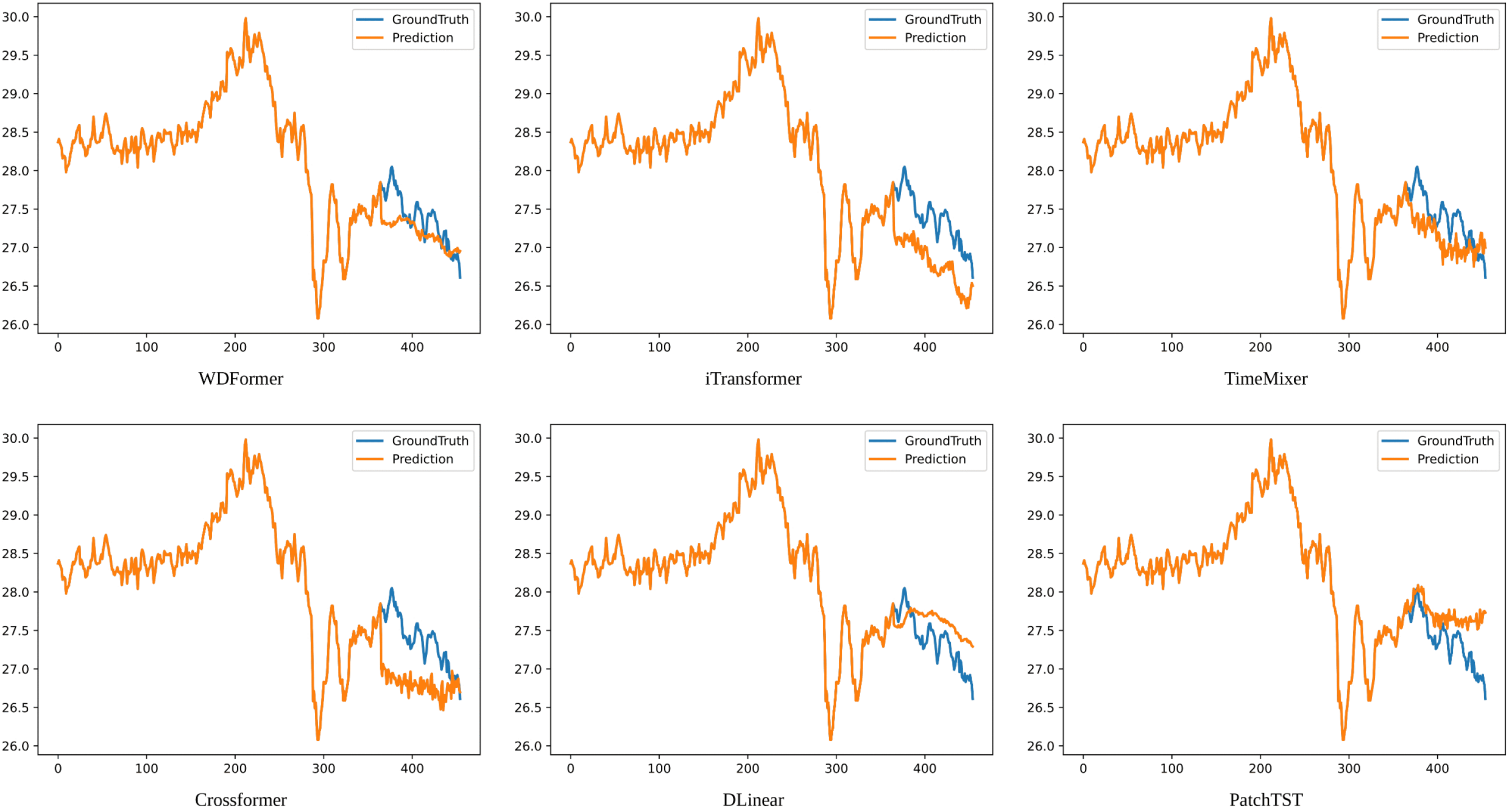
90-day prediction comparison for the 5N110W dataset.

**Fig 5:**
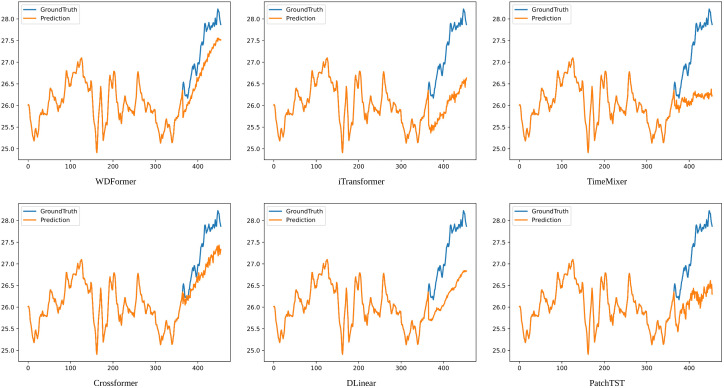
90-day prediction comparison for the 0N155W dataset.

**Fig 6:**
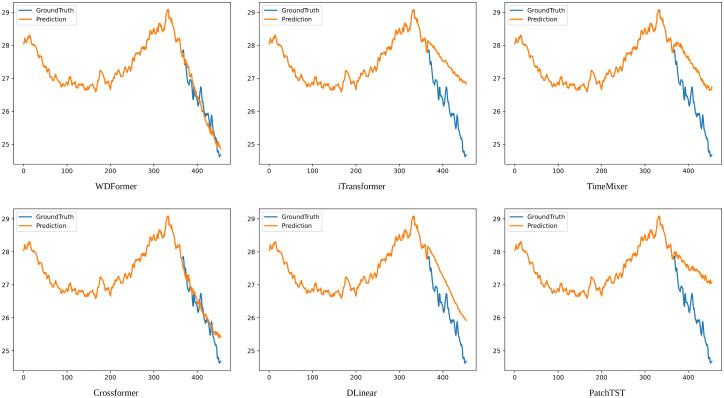
90-day prediction comparison for the 5S125W dataset.

**Fig 7:**
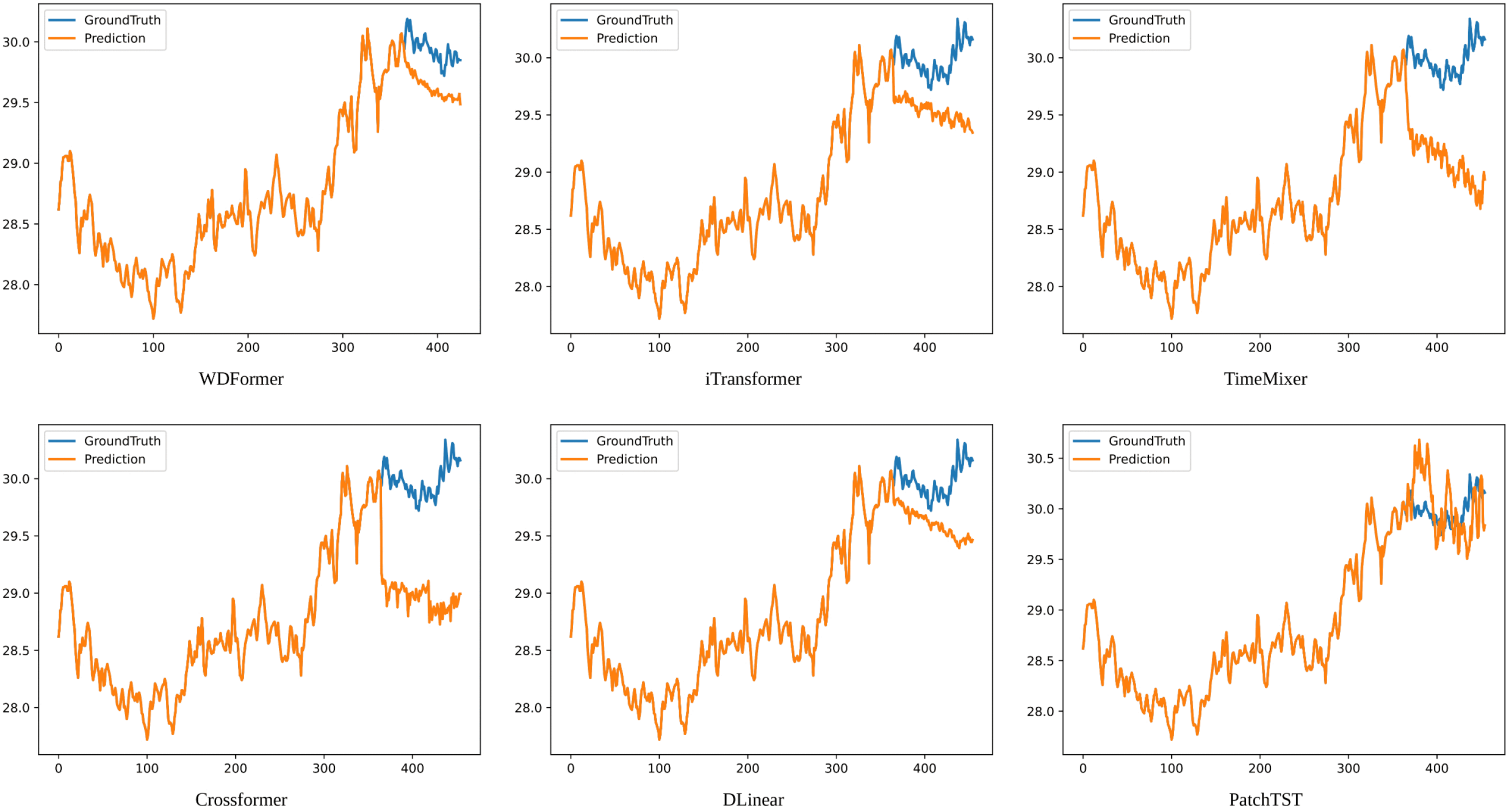
90-day prediction comparison for the 5S180W dataset.

Figs 3–7 show 90-day SST prediction comparisons for WDFormer and all five baseline models across the five datasets. All models use a 365-day look-back window. The blue line represents the ground truth and the orange line represents the model prediction. WDFormer generally follows the true series more closely in both slowly varying patterns and short-term variability, which is consistent with the quantitative results.

### Ablation studies

To validate each component, we evaluate four ablation variants: without wavelet decomposition (w/o Decomp), without the original path (w/o Orig), without the trend path (w/o Trend), and without the residual path (w/o Resid). Table 5 presents full results.

**Table 5:** Ablation study results (RMSE / MAE) across all datasets. Best results per scenario are bold.

Dataset Horizon		WDFormer		w/o Decomp		w/o Orig		w/o Trend		w/o Resid	
		RMSE	MAE	RMSE	MAE	RMSE	MAE	RMSE	MAE	RMSE	MAE
9N140W	7	0.238	0.187	0.257	0.201	0.264	0.209	0.240	0.189	**0.231**	**0.179**
	15	0.310	0.245	0.331	0.260	0.323	0.255	0.312	0.247	**0.302**	**0.236**
	30	**0.354**	**0.278**	0.381	0.302	0.365	0.290	0.357	0.284	0.373	0.294
	60	**0.408**	**0.330**	0.473	0.383	0.421	0.341	0.411	0.334	0.438	0.353
	90	**0.444**	**0.359**	0.518	0.427	0.457	0.373	0.445	0.362	0.475	0.389
5N110W	7	0.329	0.252	0.360	0.273	0.366	0.274	0.330	0.253	**0.325**	**0.250**
	15	**0.429**	**0.327**	0.481	0.369	0.456	0.341	0.433	0.333	0.430	0.328
	30	**0.495**	**0.377**	0.548	0.426	0.515	0.393	0.498	0.381	0.509	0.389
	60	**0.577**	**0.445**	0.657	0.514	0.602	0.462	0.593	0.459	0.633	0.488
	90	**0.643**	**0.495**	0.698	0.538	0.671	0.508	0.661	0.502	0.722	0.559
0N155W	7	**0.323**	**0.245**	0.394	0.294	0.365	0.277	0.329	0.249	0.338	0.254
	15	**0.406**	**0.305**	0.506	0.385	0.409	0.308	0.416	0.315	0.430	0.324
	30	**0.462**	**0.354**	0.582	0.447	0.471	0.359	0.465	0.359	0.508	0.387
	60	**0.538**	**0.423**	0.625	0.474	0.556	0.435	0.540	0.424	0.582	0.453
	90	**0.569**	**0.450**	0.687	0.533	0.601	0.477	0.574	0.453	0.629	0.496
5S125W	7	**0.251**	**0.181**	0.289	0.220	0.268	0.198	0.270	0.204	0.274	0.203
	15	**0.330**	**0.242**	0.399	0.308	0.345	0.258	0.362	0.272	0.364	0.277
	30	0.379	0.282	0.415	0.321	**0.370**	**0.276**	0.382	0.287	0.428	0.333
	60	**0.433**	**0.337**	0.472	0.370	0.449	0.361	0.441	0.348	0.496	0.396
	90	0.459	**0.362**	0.558	0.441	0.466	0.368	**0.458**	0.362	0.537	0.425
5S180W	7	0.234	0.182	0.264	0.208	0.246	0.196	**0.233**	**0.180**	0.236	0.182
	15	**0.273**	**0.217**	0.314	0.247	0.283	0.224	0.292	0.235	0.312	0.254
	30	**0.327**	**0.262**	0.335	0.267	0.337	0.274	0.328	0.265	0.341	0.275
	60	**0.397**	**0.326**	0.424	0.346	0.405	0.335	0.398	0.328	0.430	0.348
	90	**0.461**	**0.373**	0.521	0.421	0.466	0.385	0.474	0.390	0.509	0.406

Removing decomposition causes the most severe degradation: at 0N155W, average RMSE increases from 0.460 to 0.559 (+21.5%), confirming that the decompose-then-model strategy is critical for handling mixed-frequency SST sequences. Removing the residual path (w/o Resid) also substantially degrades performance (0N155W: RMSE increases to 0.497), underlining the importance of a dedicated Transformer for high-frequency dynamics. Removing the original path shows that preserving unfiltered information compensates for potential decomposition losses. Removing the trend path confirms that isolating low-frequency modeling frees the Transformer to focus on complex residual dynamics.

### Sensitivity to look-back window

**Fig 8:**
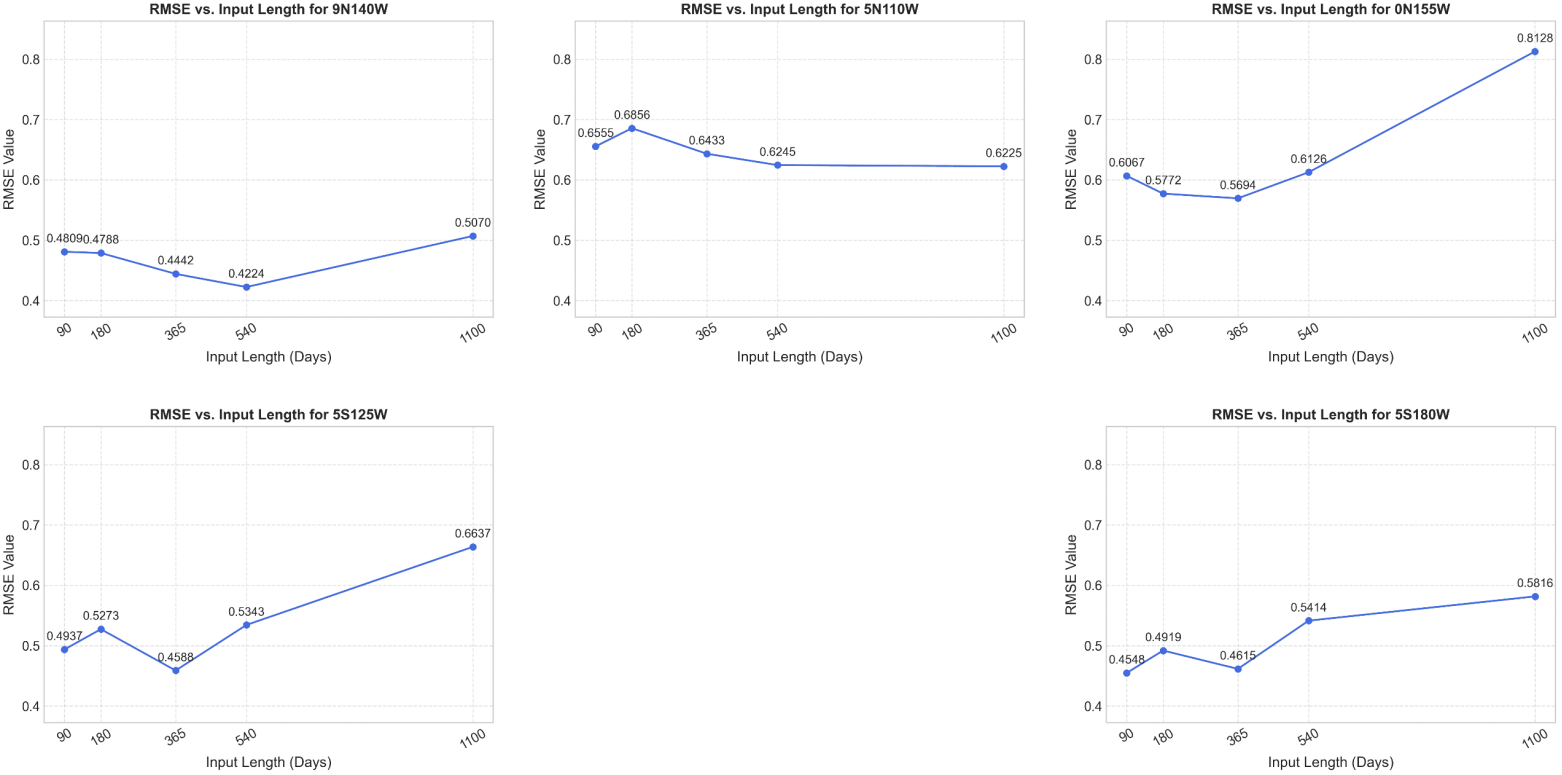
Sensitivity of WDFormer to look-back window size. RMSE for the 90-day prediction horizon is shown as a function of input window length (days) for each of the five datasets.

We evaluated WDFormer on the 90-day task with five look-back windows: 90, 180, 365, 540, and 1100 days (Fig 8). The optimal window varied across stations. A 365-day window achieved the lowest RMSE at 0N155W (0.569) and 5S125W (0.459), suggesting that including a complete annual cycle can help the model capture seasonal SST evolution at these locations. Longer windows were more effective at 9N140W (540 days, RMSE 0.422) and 5N110W (1100 days, RMSE 0.623), indicating that extended historical context may provide useful information for these stations. In contrast, 5S180W achieved its lowest RMSE with a 90-day window (0.455), suggesting that recent SST evolution may be more informative than distant history for this particular prediction task.

Overall, these results show that the look-back window should be treated as a station- and task-dependent hyperparameter. Its optimal value depends on local SST variability, the forecast horizon, and the model’s feature-learning behavior, and therefore should be selected empirically rather than fixed a priori.

### ENSO-phase and El Niño stage stratified evaluation

To assess WDFormer’s skill under different ENSO-related background states, we performed an ONI-based stratified evaluation for the 90-day task. ENSO phases were classified using the NOAA Climate Prediction Center Oceanic Niño Index (ONI), a three-month running mean of Niño 3.4 SST anomalies, with a ±0.5°C threshold. Each 90-day forecast sample was assigned to the dominant monthly ENSO phase within its target window. This procedure yielded 1,830 El Niño, 2,495 La Niña, and 1,435 Neutral samples across the five datasets.

Table 6 reports the sample-weighted RMSE, MAE, sample count, and 95% bootstrap uncertainty for each ONI-based phase. To improve readability, the RMSE confidence intervals are reported as point estimates with symmetric bootstrap half-widths. WDFormer achieved the lowest RMSE under all three phases. The advantage was largest during El Niño conditions, where WDFormer obtained an RMSE of 0.454±0.008, compared with 0.534 for the second-best model, PatchTST. Under La Niña conditions, WDFormer also achieved the lowest RMSE (0.509±0.009), although the margin over iTransformer was smaller. Under Neutral conditions, WDFormer obtained the lowest point estimate of RMSE, but the improvement over DLinear was modest. These results indicate that the decomposition-based heterogeneous architecture provides its clearest additional benefit during warm-event conditions, while still remaining competitive in other ENSO-related background states.

**Table 6:** Sample-weighted RMSE and MAE by ONI-based ENSO phase for the 90-day task. Uncertainty is reported as the point RMSE ± the 95% bootstrap half-width. Results are aggregated across all five datasets. Best RMSE values are shown in bold. Entries are reported as RMSE ± half-width / MAE.

Model	El Niño	La Niña	Neutral
WDFormer	**0.454 ± 0.008 / 0.370**	**0.509 ± 0.009 / 0.404**	**0.615 ± 0.017 / 0.466**
iTransformer	0.574 ± 0.013 / 0.468	0.529 ± 0.009 / 0.420	0.789 ± 0.020 / 0.613
PatchTST	0.534 ± 0.011 / 0.423	0.587 ± 0.011 / 0.458	0.823 ± 0.021 / 0.625
Crossformer	0.690 ± 0.016 / 0.546	0.616 ± 0.014 / 0.476	0.697 ± 0.018 / 0.535
DLinear	0.688 ± 0.015 / 0.566	0.552 ± 0.009 / 0.434	0.641 ± 0.019 / 0.494
TimeMixer	0.589 ± 0.012 / 0.468	0.600 ± 0.012 / 0.466	0.735 ± 0.018 / 0.572
Count	1,830	2,495	1,435

**Fig 9:**
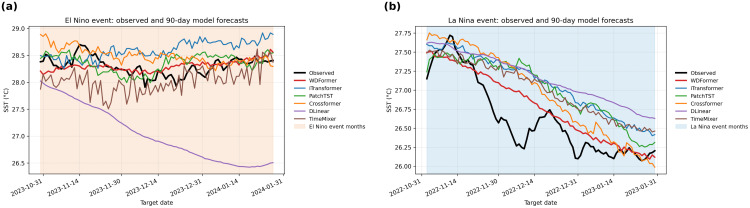
Observed SST and 90-day model forecasts during a representative El Niño/La Niña event.

Representative event-window forecasts provide a complementary view of the phase-stratified metrics. Fig 9(a) shows an El Niño example at 5N110W. The observed SST remains persistently warm but contains short-lived fluctuations. WDFormer follows the observed trajectory more closely than most baselines and avoids the pronounced cold drift seen in DLinear and the late warm bias in iTransformer. PatchTST and TimeMixer capture part of the low-frequency evolution, but their departures from the observed curve are larger during several target periods. This example is consistent with the stronger El Niño-phase advantage reported in Table 6.

Fig 9(b) shows a La Niña example at 9N140W during the boreal winter target window. The observed curve contains a rapid cooling phase followed by partial recovery and renewed cooling. WDFormer captures the overall cooling evolution more effectively than the smoother baselines, although it still underestimates some day-to-day variability. Crossformer follows the decline but tends to become too cold near the end of the window, whereas DLinear and several Transformer baselines show a smoother, warmer trajectory. Together, the two event examples indicate that WDFormer improves the event-scale SST trajectory while preserving the main limitations of deterministic 90-day point forecasts.

Because averaged ENSO-phase metrics may still mask differences among event stages, we further divided El Niño months into onset/development, mature/peak, and decay/termination stages. Continuous El Niño events were first identified from ONI months exceeding the 0.5°C threshold. For each event, the month with the maximum ONI was identified as the peak month. The peak month and its immediate neighboring months were labeled as the mature/peak stage, months before this stage were labeled as onset/development, and months after this stage were labeled as decay/termination. Each 90-day forecast sample was then assigned to the dominant El Niño stage within its target window.

The stage-wise results in Table 7 show that WDFormer achieved the lowest RMSE in all three El Niño stages. The model obtained RMSE values of 0.589±0.015 during onset/development, 0.358±0.013 during mature/peak, and 0.433±0.019 during decay/termination. The largest absolute error occurred during onset/development, suggesting that early-stage El Niño evolution remains the most challenging regime. Nevertheless, WDFormer maintained a clear advantage across the three stages, indicating that the proposed decomposition-based architecture remains effective not only under different ENSO phases but also across the main stages of El Niño event evolution.

**Table 7:** El Niño stage-wise RMSE and MAE for the 90-day task. Uncertainty is reported as the point RMSE ± the 95% bootstrap half-width. Results are aggregated across all five datasets. Best RMSE values are shown in bold. Entries are reported as RMSE ± half-width / MAE.

Model	Onset/development	Mature/peak	Decay/termination
WDFormer	**0.589 ± 0.015 / 0.487**	**0.358 ± 0.013 / 0.285**	**0.433 ± 0.019 / 0.331**
iTransformer	0.771 ± 0.020 / 0.647	0.478 ± 0.018 / 0.387	0.489 ± 0.020 / 0.372
PatchTST	0.722 ± 0.021 / 0.577	0.452 ± 0.010 / 0.362	0.547 ± 0.021 / 0.405
Crossformer	0.765 ± 0.020 / 0.620	0.669 ± 0.036 / 0.510	0.655 ± 0.022 / 0.539
DLinear	0.736 ± 0.021 / 0.608	0.870 ± 0.029 / 0.735	0.533 ± 0.018 / 0.438
TimeMixer	0.726 ± 0.016 / 0.604	0.465 ± 0.017 / 0.370	0.564 ± 0.023 / 0.403
Count	1,145	460	670

## Discussion

### Scope of evaluation and generalizability

The evaluation is conducted using five representative local-grid OISST subregions. WDFormer outperforms the tested deep learning baselines at most 30–90-day horizons, but the short-horizon results also show that persistence remains a strong baseline at 7 days, reflecting the high autocorrelation of daily SST. Because the current evaluation is based on selected local grids, the results should be interpreted as evidence for regional daily SST forecasting; broader spatial generalization should be examined in future gridded extensions.

Direct comparison with operational dynamical prediction systems (e.g., NMME or ECMWF S2S) is not straightforward in this framework because those systems provide monthly or seasonal forecasts with different initialization conventions and verification procedures [16]. Persistence and daily climatology are therefore used as matched reference baselines under the same daily verification protocol.

### Physical interpretation of the decomposed components

The decomposed components in WDFormer should be interpreted as data-driven frequency bands rather than direct dynamical modes. The low-frequency branch mainly represents slowly varying background SST and seasonal-scale evolution, which may be related to the persistent thermal state of the upper ocean. The residual branch emphasizes departures from this smooth background, including intraseasonal variability, short-lived air–sea perturbations, and unresolved local noise. This interpretation is consistent with the architecture: the smooth component is handled by a lightweight MLP, whereas the irregular component is assigned to a Transformer branch with stronger capacity for nonlinear dependency modeling. However, without subsurface temperature, wind stress, mixed-layer information, or current fields, the present study cannot attribute these components to specific oceanic processes. Future multi-variable extensions may help connect the learned components more directly with known ENSO-related mechanisms and background climate variability [1,4,13].

### Limitations

**Forecast uncertainty.** WDFormer currently produces deterministic point forecasts. Probabilistic extensions, such as Monte Carlo Dropout, quantile regression, ensemble forecasting, or conformal prediction, would provide useful uncertainty estimates for future applications.

**Climate non-stationarity.** The model is trained on 1991–2024 data, a period during which SST baselines and ENSO characteristics may have shifted under anthropogenic warming. Learned statistical relationships may therefore be affected by future changes in SST trends and variability. Mitigation strategies include periodic retraining, sliding-window updating, and domain adaptation.

**Single-variable input.** Only daily SST is used as input. Incorporating additional variables—sea surface height, chlorophyll-*a*, mixed layer depth, wind stress—could improve physical relevance and forecasting skill.

**Connection to downstream applications.** The 7–90-day SST forecasts could provide short-lead environmental covariates for habitat suitability models, fish distribution models, coral bleaching alerts, or aquaculture risk monitoring. For example, predicted SST anomalies could be converted into threshold-based warning indicators or used as input features in ecological forecasting models. Coupling WDFormer with such application models remains a natural direction for future work.

## Conclusion

We proposed WDFormer, a wavelet-based heterogeneous three-path fusion model for daily SST forecasting in ENSO monitoring regions. WDFormer separates the input sequence into smoother low-frequency and higher-frequency residual components, assigning dedicated processing branches to each. Experiments on five representative OISST datasets (1991–2024) show that WDFormer performs competitively against five deep learning baselines, with clearer advantages at 30–90-day horizons. Persistence outperforms WDFormer at 7 days but degrades rapidly with lead time; WDFormer reduces average RMSE relative to persistence by 10.7%, 23.2%, and 31.9% at 30, 60, and 90 days, respectively. The ONI-based ENSO-phase and El Niño-stage stratified evaluations confirm that WDFormer achieves the lowest RMSE under El Niño, La Niña, and Neutral conditions, as well as during El Niño onset/development, mature/peak, and decay/termination stages for the 90-day task. Additional bootstrap analyses support the robustness of WDFormer at the 90-day horizon, and ablation studies confirm the contribution of wavelet decomposition and the heterogeneous multi-path design.

Future work will target: (i) probabilistic forecasting via quantile regression, ensemble modeling, or conformal prediction; (ii) multi-source environmental inputs; (iii) broader spatial extension; and (iv) periodic retraining and domain adaptation to address climate non-stationarity.
